# Micrometer-resolution imaging using MÖNCH: towards G_2_-less grating interferometry

**DOI:** 10.1107/S1600577516014788

**Published:** 2016-10-17

**Authors:** Sebastian Cartier, Matias Kagias, Anna Bergamaschi, Zhentian Wang, Roberto Dinapoli, Aldo Mozzanica, Marco Ramilli, Bernd Schmitt, Martin Brückner, Erik Fröjdh, Dominic Greiffenberg, Davit Mayilyan, Davide Mezza, Sophie Redford, Christian Ruder, Lukas Schädler, Xintian Shi, Dhanya Thattil, Gemma Tinti, Jiaguo Zhang, Marco Stampanoni

**Affiliations:** a Paul Scherrer Institute, 5232 Villigen PSI, Switzerland; b Institute for Biomedical Engineering, University and ETH Zurich, 8092 Zurich, Switzerland

**Keywords:** hybrid detectors, silicon detectors, interpolation, grating interferometry

## Abstract

MÖNCH is a 25 µm-pitch charge-integrating detector aimed at exploring the limits of current hybrid silicon detector technology. The small pixel size makes it ideal for high-resolution imaging. With an electronic noise of about 110 eV r.m.s., it opens new perspectives for many synchrotron applications where currently the detector is the limiting factor, *e.g.* inelastic X-ray scattering, Laue diffraction and soft X-ray or high-resolution color imaging. Due to the small pixel pitch, the charge cloud generated by absorbed X-rays is shared between neighboring pixels for most of the photons. Therefore, at low photon fluxes, interpolation algorithms can be applied to determine the absorption position of each photon with a resolution of the order of 1 µm. In this work, the characterization results of one of the MÖNCH prototypes are presented under low-flux conditions. A custom interpolation algorithm is described and applied to the data to obtain high-resolution images. Images obtained in grating interferometry experiments without the use of the absorption grating G_2_ are shown and discussed. Perspectives for the future developments of the MÖNCH detector are also presented.

## Abstract 2

The MÖNCH 25 µm-pitch hybrid pixel detector is described in detail and characterized. The interpolation algorithm developed to achieve micrometer-level resolution is applied to grating interferometry measurements.

## 1. Introduction   

Hybrid pixel detectors are widely used in X-ray applications as they are able to fulfill most of the requirements of the experiments: single-photon sensitivity, large dynamic range, wide area coverage, fast frame rate, simple, stable and user-friendly operation.

Photon-counting detectors are well established at synchrotrons, *e.g.* PILATUS (Kraft *et al.*, 2009 ▸), EIGER (Dinapoli *et al.*, 2011 ▸), MEDIPIX (Gimenez *et al.*, 2015 ▸), IMXPAD (Medjoubi *et al.*, 2012 ▸). However, due to the pulsed structure of the beam, they are unusable at X-ray free-electron lasers (XFELs). In the last few years, this boosted the development of charge-integrating hybrid detectors like CSPAD (Herrmann *et al.*, 2014 ▸), GOTTHARD (Mozzanica *et al.*, 2012 ▸), AGIPD (Greiffenberg, 2012 ▸), DSSC (Porro *et al.*, 2012 ▸), LPD (Koch *et al.*, 2013 ▸), JUNGFRAU (Mozzanica *et al.*, 2014 ▸). These detectors can offer the same data quality as photon-counting detectors (Henrich *et al.*, 2011 ▸), while overcoming some of their disadvantages, including the minimum detectable energy, the saturation at high count rates and the limits on the pixel size due to charge sharing (Bergamaschi *et al.*, 2014 ▸).

However, hybrid detectors have never been considered as candidates for soft X-ray detection due to their relatively large electronic noise [a few hundreds eV r.m.s. compared with a few tens eV r.m.s. offered by drift detectors (Quaglia *et al.*, 2015 ▸)]. Only recently has it been shown, by Jungmann-Smith *et al.* (2016 ▸), that hybrid detectors can offer an energy resolution better than 100 eV r.m.s. and can be used for X-ray experiments down to 1 keV or for energy-dispersive imaging.

In addition, the bump-bonding technique necessary to connect the sensor to the front-end electronics has always set a limit on the minimum pixel size of ∼50 µm, preventing hybrid detectors from being used for high-resolution imaging.

MÖNCH is a charge-integrating detector which aims to overcome the main limitations of the current hybrid detector technology, focusing in particular on the high spatial resolution provided by the 25 µm-pitch pixels. MÖNCH is optimized for low noise, but, despite the small area available for the pixel electronics and the limitations of the power consuption per pixel, it still has a relatively high dynamic range considering the 25 µm pitch (Dinapoli *et al.*, 2014 ▸).

With high frame rates and moderate photon fluxes, the detector can operate in the single-photon regime (*i.e.* with less than one photon detected on average per 3 × 3 pixels), discriminating single photons from the electronic noise and separating them spatially. In this operation mode, it delivers the same data quality as photon-counting detectors but with a pixel pitch at which photon-counting detectors could not operate due to the high level of charge sharing. Additionally, the analog readout provides spectral information concerning the detected X-rays.

Many applications can benefit from the spatial resolution which can be achieved already thanks to the 25 µm pixel pitch. This, coupled to the outstanding low noise, allows also soft X-ray or energy-dispersive imaging with an energy-resolving power mainly limited by the electronic and by the Fano noise.

Moreover, the low noise, together with the large amount of charge sharing, permits the absorption position of the photons to be estimated with sub-pixel resolution using interpolation (Schubert *et al.*, 2012 ▸). The micrometer-level spatial resolution makes MÖNCH ideal for high-resolution imaging techniques.

In the following, we discuss the applicability of MÖNCH to single-shot grating interferometry (Wen *et al.*, 2008 ▸), a radiographic technique whose application to clinical practice is limited by the relatively large pixel size of state-of-the-art medical imaging detectors (usually ≥20 µm). In fact, in order to directly resolve the fringes generated by the analyzer grating (G_1_) and the phase shifts introduced by the samples, the spatial resolution must be significantly smaller than the few micrometers period of G_1_. Therefore, in Talbot–Lau grating interferometry (Pfeiffer *et al.*, 2006 ▸), an absorption grating (G_2_) with a pitch matching the period of the fringes generated by G_1_ is normally stepped in front of the detector to resolve the sub-microradians fringes of G_1_ on larger pixel detectors (Weitkamp *et al.*, 2005 ▸). This translates into a low dose efficiency (due to the absorption of 50% of the X-rays by G_2_), increased measurement times, challenging mechanical stability and difficulties in fabricating large-area gratings for hard X-rays (Roessl *et al.*, 2014 ▸).

The micrometer-level resolution delivered by the MÖNCH detector after interpolation allows the interference fringes to be resolved without the use of G_2_. The phase shift introduced by the sample can be retrieved from the MÖNCH data by combining position interpolation algorithms with an algorithm based on a Hilbert transform optimized to compensate for the position-dependent spatial resolution of the detector.

In this work, §2 describes the MÖNCH detector system and the MÖNCH0.2 prototype. §3 shows the characterization measurements. The data analysis and algorithms for position interpolation of single photons are explained in detail and applied to the imaging of a biological sample in §4. In §5 the proof of principle of a G_2_-less grating interferometry experiment is demonstrated. Finally, the results are discussed and perspectives for future optimization are given.

## 2. MÖNCH detector description   

### 2.1. The MÖNCH hybrid detector   

MÖNCH is a charge-integrating hybrid pixel detector project with a small pixel pitch of 25 µm currently developed at the Paul Scherrer Institut (PSI, Switzerland) (Dinapoli *et al.*, 2014 ▸). The sensor consists of a 320 µm-thick n-doped high-resistivity silicon wafer. The n^+^-doped back-plane is kept at a stable high-bias voltage of 90–120 V, while the 25 µm-pitch p^+^-doped electrodes are connected to the readout electronics by means of indium bumps of size a few micrometers (Lutz, 1999 ▸).

The X-rays are absorbed in the silicon sensor producing electron–hole pairs (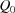 = *E*_0_/3.62 eV in silicon, where 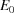 is the photon energy and 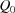 is the number of electron–hole pairs generated). Due to the high electric field applied to the fully depleted silicon wafer, the electrons drift towards the back-plane, while the holes are collected by the p^+^ implants and are then integrated in parallel and fully independently by each single pixel in the Application Specific Integrated Circuit (ASIC), which is read out over several serial analog lines and finally digitized by external commercial analog-to-digital converters (ADCs).

While drifting to the collecting electrodes, the charge cloud diffuses and can be collected by several pixels, depending on the absorption position. This effect is known as charge sharing and is more prominent in smaller pixel pitch detectors. The amount of charge sharing depends on several parameters including the sensor thickness, the sensor bias and the photon energy (Cartier *et al.*, 2014 ▸, 2015 ▸). It has been measured that, for a 320 µm-thick silicon sensor biased with 120 V, the size of the charge cloud is of the order of 17 ± 3 µm in the 10–20 keV energy range (Bergamaschi *et al.*, 2008 ▸).

The 25 µm pixel pitch has been chosen as a trade-off between opposite constraints. On the one hand the pitch has to be small enough such that the charge produced by the majority of the photons will be shared between neighboring pixels to be able to effectively perform interpolation. On the other hand, small pixel sizes are very challenging both in terms of bump-bonding yield, due to the small size and increasing force to be applied, and in terms of electronics design due to the constraints in the pixel area and power consumption.

ASIC and sensor design as well as bump-bonding are performed in-house by the Swiss Light Source (SLS) Detector group.

In this commissioning phase, the same readout board as developed for the GOTTHARD microstrip detector has been used for data acquisition (Mozzanica *et al.*, 2012 ▸), with some adaptation of the firmware and software to match the requirements of the MÖNCH pixel detector. With a 1 Gb s^−1^ data transfer interface, it allows a maximum frame rate of 1 kHz.

### 2.2. The MÖNCH readout chip   

Several prototype ASICs were designed in UMC 110 nm technology. Details can be found in the paper by Dinapoli *et al.* (2014 ▸). The data presented in this work have been acquired using the MÖNCH0.2 prototype. It is a fully functional small-scale ASIC of 4 mm × 4 mm, containing an array of 160 × 160 pixels. This array is subdivided into five blocks, each featuring a different pixel architecture. Two blocks have statically selectable preamplifier gains and target synchrotron applications. In low-gain mode they still provide single-photon sensitivity for energies higher than 6 keV as well as a reasonable dynamic range for such a small area (>120 12 keV photons). In high gain, they target high-resolution low-flux experiments where charge sharing can be exploited to reach micrometer-level resolution. Three other architectures address possible uses at XFELs and implement automatic switching between two gains to increase the dynamic range, as well as input overvoltage control.

The dynamic range of charge-integrating detectors scales with the available area for the integration capacitance in the pixel. Therefore compared with a larger pixel pitch of 75 µm (*e.g.* JUNGFRAU) the dynamic range per pixel is reduced by about a factor of nine, but remains constant per unit of area, allowing the same total flux to be measured.

**Figure 1:**
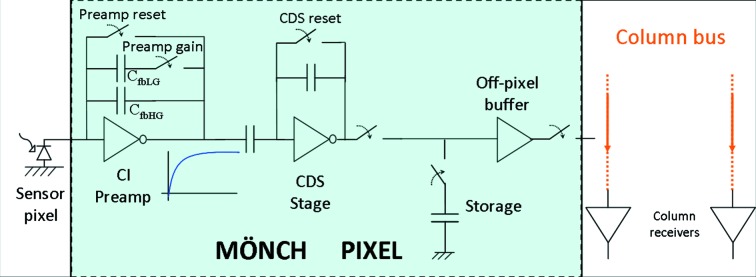
Simplified diagram of the basic pixel architecture of MÖNCH.

The basic pixel structure, common to all sub-blocks, is shown in Fig. 1 ▸. The charge produced in the sensor by the impinging photons is integrated by the feedback capacitor of the charge amplifier. Two different capacitors can be switched into the feedback loop to obtain two different preamp gains. A correlated double sampling (CDS) stage follows, to reduce the low-frequency noise contributions coming from the preamp and its reset transistor (Buttler *et al.*, 1990 ▸). The output of the CDS is stored locally on a capacitor, which gives the pixel the ability to be continuously sensitive: after storage the pre­amplifier and CDS are available again for processing the next image, while the readout of the current image can happen simultaneously. The gain of the CDS buffer can be statically selected between 4 (for low-noise applications) and 0.5 (to extend the dynamic range).

The voltage stored on the storage capacitor is driven to the chip periphery by an off-pixel buffer and is refreshed by a column buffer. The signal produced by every column buffer is serially multiplexed to a common single-ended-to-differential off-chip buffer (not shown in the figure).

All the results shown in this paper are obtained using a single sub-block of 40 × 160 pixels (1 mm × 4 mm) of MÖNCH0.2 optimized for single-photon sensitivity by using the high preamplifier gain and a CDS gain of 4. The same pixel architecture was also selected for the design of MÖNCH0.3, a 10 mm × 10 mm (400 × 400 pixels) chip, which is at present undergoing test and characterization.

### 2.3. Cluster finding   

Due to charge sharing, a single pixel only partially collects the charge generated by a photon. Therefore, the summation of the charge from the cluster of channels among which it is shared (clustering) is required to retrieve the correct radiation spectrum. To analyze single-photon absorption events which are shared between neighboring pixels, a cluster finding algorithm (CFA) has been developed, as described by Cartier *et al.* (2014 ▸). The CFA is effective only on datasets with low occupancy, *i.e.* with on average less than one photon per 3 × 3 pixel cluster (single-photon regime). It considers as photons only the events where either the total signal collected by a cluster or the signal of a single pixel exceed the electronic noise by five times its electron noise charge (ENC), defined as the signal at the input of the electronic chain which would result in the measured noise (Radeka, 1988 ▸). The pixel pedestal and the electronic noise threshold are continuously tracked during the acquisition to compensate for drifts induced in the dark image signal and noise properties of each pixel by temperature and other environmental changes. Additional constraints for adjacent pixels are applied to ensure that only one cluster is extracted per photon hit and overlaps of clusters from more than one photon are discarded. A good signal-to-noise ratio (SNR) is crucial in order to detect also the photons for which the charge is shared and collected by several pixels in the cluster.

**Figure 2:**
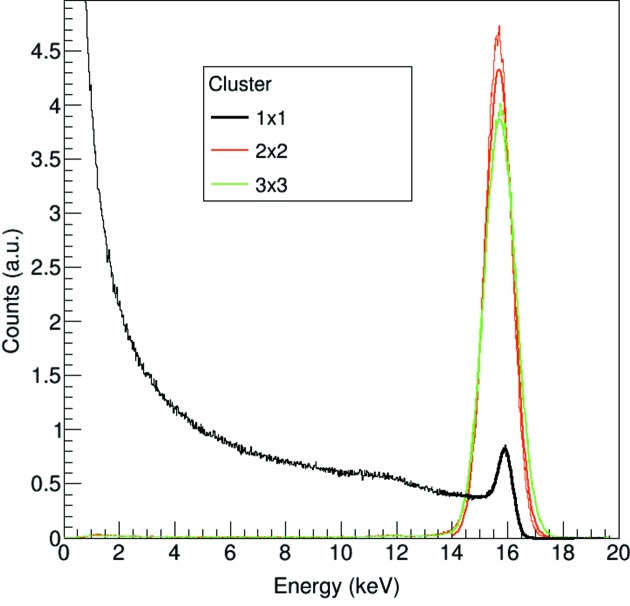
Spectrum of a single pixel [fitted using equation (1)], 2 × 2 pixel and 3 × 3 pixel clusters (fitted with a Gaussian) acquired at 16 keV.

Fig. 2 ▸ shows the spectrum of 16 keV monochromatic radiation for a single pixel and for 2 × 2 and 3 × 3 pixels. While a single pixel carries only limited information concerning the X-ray energy, the full charge is already retrieved by a 2 × 2 pixel cluster, despite the increase in noise by a factor of two due to summation.

§4 explains in detail how it is possible to improve the image resolution beyond the pixel size by analyzing the charge ratio between the individual pixels of each cluster. The final resolution depends not only on the amount of charge sharing but also on the SNR, hence the effort on limiting the electronic noise in the MÖNCH ASIC.

## 3. MÖNCH characterization   

The MÖNCH0.2 ASIC bump-bonded in-house to 160 × 160 25 µm-pitch pixels, 320 µm-thick silicon sensor produced by HAMAMATSU, has been thoroughly characterized. Here we report the major achievements regarding the first supercolumn of the ASIC operated in the single-photon regime which has been used for the imaging experiments shown in §§4 and 5

### 3.1. Bump-bond yield   

Given the small pixel pitch, the feasibility of the bump-bonding technique developed and applied in-house at PSI had to be demonstrated. The process required only minor modifications compared with the one used for large pixels (*e.g.* PILATUS 172 µm, EIGER 75 µm). The size of the under-bump-metallization and indium bumps have been adapted to the 25 µm pitch for the processing of the ASIC and of the sensor wafers. The pressure applied during the bump-bonding procedure also had to be rescaled to compensate for the much higher pixel density.

**Figure 3:**
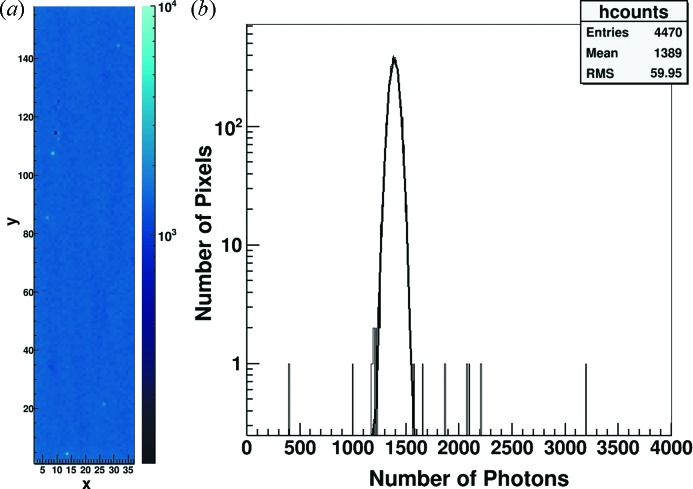
(*a*) Flat-field image and (*b*) count distribution of the first supercolumn of MÖNCH0.2. Only two of the 4470 pixels plotted count too few photons and can be attributed to faulty bump-bonding. The estimated bump-bond yield is better than 99.95%.

Fig. 3(*a*) ▸ shows an image of a flat-field and Fig. 3(*b*) ▸ represents the count distribution acquired in the single-photon regime at 16.7 keV at the TOMCAT beamline of the SLS after applying the CFA for the first supercolumn of the detector assembly used for the measurements in this paper. The border pixels have been excluded from the analysis because clusters are lost along the edges.

The photon distribution is uniform over the whole detector taking into account the variations of the illuminating beam and the gain differences between pixels. Only the two pixels with too few counts can be attributed to faulty bump-bonding, resulting in a bump-bond yield of better than 99.95%. Comparable results have been obtained also on the other detectors assembled. During the development phase, the main issues came from the processing of the single ASIC dice since full wafers cannot be purchased in the prototyping phase. The bump-bond yield is close to this level also for the larger 1 cm × 1 cm (400 × 400 pixels) MÖNCH0.3 detector.

The outstanding bump-bonding yield is a very important achievement in the development of small pitch pixel detectors and it is particularly crucial in the case of high-resolution imaging applications using interpolation. In fact a non-bump-bonded pixel affects also the charge collection in its neighbors and therefore prevents the use of the full 3 × 3 pixel cluster for interpolation. Moreover these nine pixels correspond to a much larger number of virtual pixels in the high-resolution rebinned image obtained after interpolation.

### 3.2. Gain calibration   

In order to extract the gain *G* necessary to convert the signal pulse height (in mV or ADC units) into energy or charge (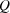 = *E*/3.6 eV), flat-field spectra at different energies need to be acquired. This calibration is necessary to correctly compare the signal collected by neighboring pixels when applying the CFA or performing interpolation (see §4).

Hereafter, the simplified linear model of charge sharing in small pitch pixel sensors described by Bergamaschi *et al.* (2015 ▸) has been used to describe the charge collection of single pixels. The spectra *S* acquired for each pixel have been fitted with the function
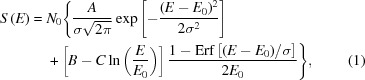
where *E* is the signal amplitude and the fit parameters are 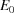 representing the X-ray energy, σ the electronic noise and 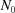 the number of photons. 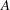, 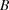, 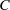 are proportional to the area where no charge sharing is observed; charge sharing occurs between two pixels (edges) and between four pixels (corners). They can be written as a function of the average size α of the charge cloud after drift and diffusion,
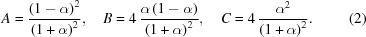
For each pixel, *G* can be extracted as the angular coefficient of the straight line correlating the X-ray energies with the parameters 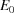 in mV or ADC units.

An average value of 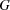 = 102.6 ± 2.9 ADC keV^−1^ has been calculated for the first supercolumn of MÖNCH0.2. The 3% spread among the channels is due to manufacturing mis-matches, but also to a reduction of the signal amplitude further away from the readout pads, due to the discharge of the storage capacitors during the readout time (droop).

**Figure 4:**
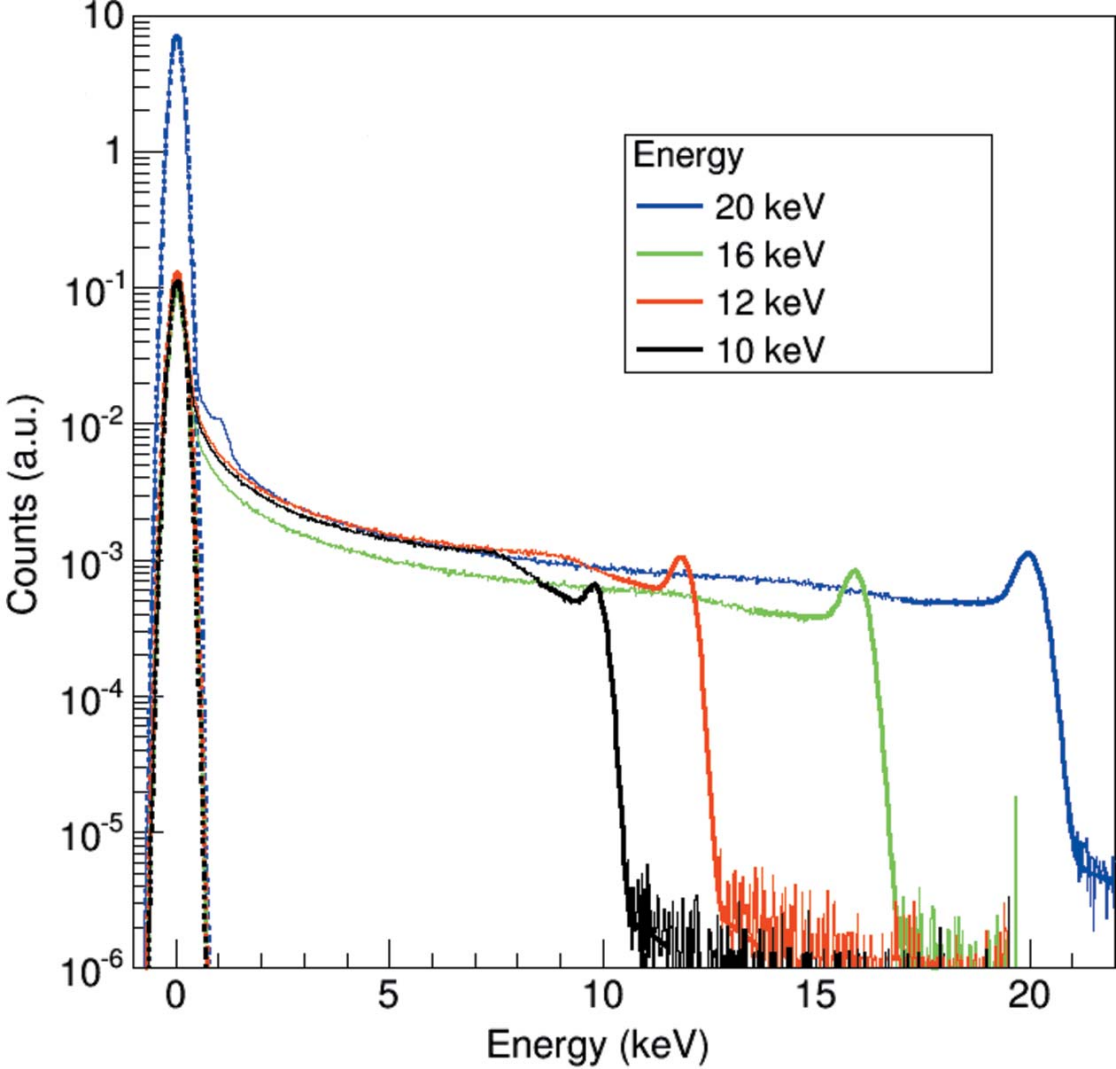
Spectrum of a single pixel at different energies. The solid line shows the fit using equation (1), while the dashed line shows the Gaussian fit of the pedestals, which can be used to estimate the electronic noise.

The cumulative spectra over all pixels of the first supercolumn at different energies fitted with equation (1) (solid line) are shown in Fig. 4 ▸. The data were acquired at the SYRMEP beamline of the Elettra synchrotron facility in Trieste, Italy (Abrami *et al.*, 2005 ▸). The goodness of the energy calibration is shown by the definition of the peaks. In the 20 keV spectrum, the Compton edge at 1.45 keV is also clearly visible close to the noise pedestal. Still, the spectra carry only limited energy information due to the large amount of charge sharing.

### 3.3. Noise   

An estimate of the electronic noise is given by the standard deviation of the dark signal peak (pedestal) of the single-pixel spectra, as shown in Fig. 4 ▸ (dashed line).

The channel-by-channel noise distribution obtained after gain calibration shows an average 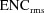 = 30 ± 3 e^−^ = 109 ± 11 eV. This spread higher than 10% is due to the discharge of the storage capacitors, causing a reduction in gain along the readout direction and therefore increasing the input noise for the pixels which are read out later in time.

The cumulative noise spectrum shows a standard deviation 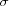 = 111 ± 1 eV after gain calibration, slightly higher than the average 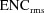 due to calibration uncertainty.

This value can be considered as the ultimate energy resolution of the detector. It allows the detection of X-rays of energy as low as 1 keV with a SNR close to 10. When summing up a cluster of 2 × 2 pixels to retrieve the full signal generated by a single X-ray, the noise increases by a factor of two, but still photons can be detected with an SNR of almost 10 already from 2 keV.

## 4. High-resolution imaging   

### 4.1. Position interpolation algorithm   

By analyzing the distribution of the charge produced by a single photon collected by a 2 × 2 pixel cluster, it is possible to extract the hit position with a resolution finer than the pixel pitch *p*.

**Figure 5:**
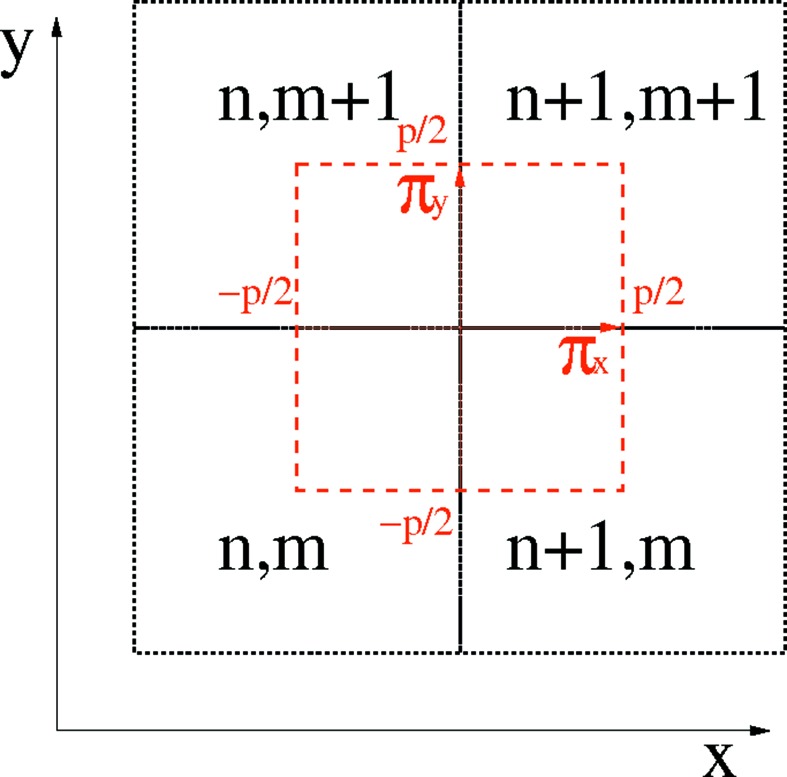
Sketch of the cluster coordinate system compared with the physical pixels. The cluster 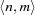 is highlighted in red. It is centered at the corner between the four physical pixels 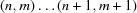 (in black) and spans between their centers. The sub-cluster coordinates 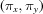 are also shown in relation to the main spatial coordinates 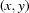.

In the following, the physical pixels are remapped into clusters 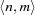 of the same pitch *p*, centered at the corner of the four pixels of the 2 × 2 pixel cluster and spanning between their centers, as shown in Fig. 5 ▸.

The distribution of the total signal 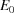 collected by the cluster 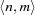 in the two Cartesian coordinates is evaluated by the parameters
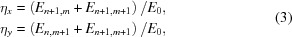
where 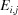 is the signal measured by the pixel at position 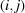 converted from the pulse height using the gain calibration as explained in §3.2.

The photons can be redistributed from η to the position space, by mapping each pair of parameters 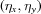 into the sub-cluster position 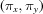 where 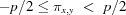.

**Figure 6:**
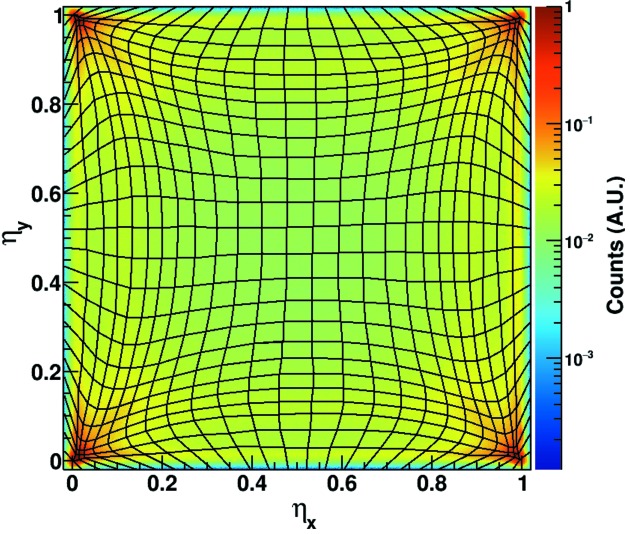
Cumulative distribution 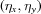 for a flat-field measurement acquired at 16.7 keV, using a 320 µm-thick sensor biased at 90 V. The rendered grid shows the partitions of the bins 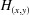 resulting from the iterative algorithm described in §4.

The main spatial position 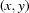 in the final image is then given by combining the cluster and the sub-cluster position,
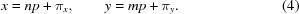
Fig. 6 ▸ shows the cumulative distribution 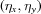 for a flat-field measurement acquired at 16.7 keV, using a 320 µm-thick sensor biased at 90 V. The maxima close to the corners of the distribution are due to the photons absorbed close to the center of the pixels (edge of the cluster), where most of the charge is collected by a single pixel due to the limited charge sharing in those areas. Since the η-distribution is not flat, a linearization algorithm has to be used in order to obtain a flat photon distribution in the sub-pixels within a cluster.

In one dimension, the non-uniform charge sharing is corrected analytically by using the so-called η-algorithm described by Belau *et al.* (1983 ▸), as shown by Schubert *et al.* (2012 ▸). A similar approach has been used by Cartier *et al.* (2014 ▸) using MÖNCH by analyzing separately the two Cartesian coordinates 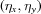.

However, due to the strong correlations between the parameters 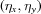 the analytical method is not sufficient to obtain a flat distribution of the photons between sub-pixels; therefore, an iterative algorithm has been developed in order to successfully solve the problem.

The goal of this method is to extract a correction map 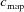 that assigns to each bin 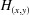 centered at 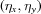 of the η-distribution histogram a sub-pixel 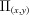 centered at 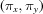 in the position space. Initially, a flat-field image needs to be acquired in order to populate the η-distribution histogram.

Since the histogram is not flat (see Fig. 6 ▸), sub-dividing the η space into bins of equal size results in a non-flat distribution of the photon hits between the sub-pixels. Therefore, the size and shape of the bins in the η histogram are adapted in order to obtain a uniform distribution [same number of entries for each 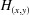 bin], which translates into a homogeneous photon density in the sub-pixels 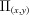, reflecting the flat illumination used.

This is obtained by iteratively adapting the position of the corners between adjacent bins in order to equalize the number of entries for each of the bins. The algorithm used relies on several boundary conditions in order to ensure full coverage of the η-space. For each iteration step, the length of the side shared between two neighboring bins is adapted linearly based on the number of entries of the bins, *i.e.* the sides of the bins with many entries are shortened while the sides of the bins with few entries are lengthened. The squared sum of the residuals of the bin entries compared with a flat illumination is used to evaluate the convergence of the algorithm at each step.

The convergence of the algorithm is assessed once the minimum and maximum deviation from the average number of counts divided by the Poisson noise are both below defined limits. This results in a flat photon distribution between the sub-pixels. An example of the partition of the bins of the η-distribution histogram is shown by the rendered grid in Fig. 6 ▸.

The resulting correction map needs to be calculated only once previous to the experiment and requires a flat-field image acquired with high statistics. Under the same experimental conditions (X-ray energy, sensor bias), 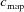 can be used to populate the virtual pixels of the high-resolution images with minimal fixed pattern noise. The ultimate spatial resolution and the possible distortions introduced in the final image using this algorithm have yet to be determined.

The spatial resolution is not uniform within the pixel due to non-linear position-dependent charge sharing. Preliminary measurements obtained by imaging an absorbing edge show a resolution better than 1 µm close to pixel corners, and up to several micrometers at the center of the pixel, where the charge is collected by a single pixel. The homogeneity of the spatial resolution can be improved by enhancing the charge sharing by increasing the drift time of the charge cloud. This can be obtained by increasing the sensor thickness, applying lower bias voltages to the sensor or detecting softer X-rays, which are absorbed closer to the sensor back-plane. Different pixel geometries to enhance the charge sharing in one direction are also being considered.

### 4.2. Imaging results   

**Figure 7:**
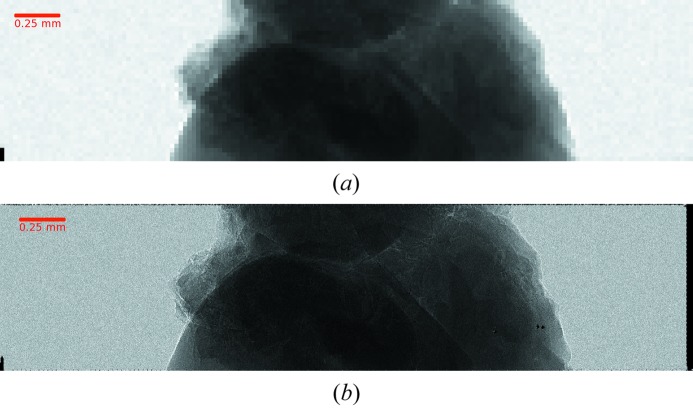
Image of a kidney stone, (*a*) 25 µm resolution image and (*b*) using 0.5 µm binning after applying the interpolation algorithm.

The improved spatial resolution obtained after interpolation has been demonstrated by acquiring radiographic images of samples containing small details. Fig. 7 ▸ shows an image of a kidney stone of diameter a few millimeters acquired at the TOMCAT beamline of the Swiss Light Source (Stampanoni *et al.*, 2007 ▸) at an energy of 16.7 keV obtained using the Si(111) monochromator and applying additional filtering to limit the photon flux and operate in the single-photon regime. Initially a flat-field image of 10.5 × 10^6^ frames with an exposure time of 12 µs each was acquired. Approximately 10 × 10^6^ photons were found by the CFA and used to populate the η-distribution and calculate the correction map for the interpolation. The image of the kidney stone sample was obtained using 10.5 × 10^6^ frames with the same exposure time and approximately 6.58 × 10^6^ photons found by the CFA.

Fig. 7(*a*) ▸ shows the image acquired by the CFA with the photon hit assigned to the 25 µm-pitch pixel collecting the maximum of the charge. The performance in terms of spatial resolution and SNR are the same as a single-photon-counter detector with charge-sharing suppression (Ballabriga *et al.*, 2007 ▸; Maj *et al.*, 2012 ▸). The image resolution is already good, but the improvements in the details of the image shown in Fig. 7(*b*) ▸ after applying the interpolation algorithm are clearly visible.

Unfortunately, the statistics per bin are quite poor; in fact, photon counts are redistributed from 40 × 160 25 µm-pitch pixels into 500 × 2000 2 µm bins, *i.e.* each virtual pixel accumulates a factor of 156 fewer photons than in the original image.

Although in this experiment the flux has not been properly optimized to acquire sufficient statistics in the shortest possible time, the acquisition time remains one of the weak points of the method, since the detector must work in the single-photon regime in order to interpolate.

In fact, even working at the maximum possible flux, one would need approximately 10 frames to acquire a photon per 25 µm-pitch pixel and 6250 frames after interpolation rebinned at 1 µm. Since the maximum frame rate of the detector is currently limited at 1 kHz, it is necessary to measure 6.25 s to acquire an image with on average 1 photon per virtual pixel. One hour of measurement would allow collection of about 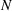 = 576 photons per virtual pixel, for SNR = 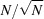 = 24. In order to detect a contrast of ∼1% it would be necessary to acquire 10000 photons per virtual pixel with a total duration of the measurement of more than 17 h at the current 1 kHz frame rate, 1.7 h at ∼10 kHz and less than 20 min at 100 kHz frame rate.

Faster frame rates could be achieved by using a 10 Gb s^−1^ instead of a 1 Gb s^−1^ transfer link, speeding up the readout by means of faster ADCs or increasing the number of analog output lines and performing real-time data compression on the readout board. This is necessary in particular for the foreseen larger area detectors in order to prevent excessive data throughput (32 GB s^−1^ cm^−2^ at 100 kHz frame rate). Event-driven readout is also a possibility which could be considered, although it introduces several issues in the data processing (*e.g.* leakage current subtraction) and is not effective in compressing the data when the occupancy is high.

The limitation in the maximum detectable flux is particularly restricting at synchrotrons, where it is impossible to make use of the huge fluxes provided by imaging beamlines, while it much better fits the low intensity generated by microfocus X-ray tubes.

## 5. G_2_-less grating interferometry   

The high position resolution obtained by interpolation is the key component for performing G_2_-less grating interferometry, since it allows the recording of changes in the position of the interference fringes from G_1_ even though the physical pixel size of the detector is much larger than the period of the fringes.

The average absorption and differential phase values are retrieved for each pixel cluster (see Fig. 5 ▸). The resulting 25 µm-pitch granularity reflects the physical size of the pixel of the MÖNCH detector and is able to satisfy the requirements of medical imaging applications like mammography, where pixel sizes of 20–50 µm are common in clinical practice (Whitman & Haygood, 2012 ▸).

### 5.1. Experimental method   

**Figure 8:**
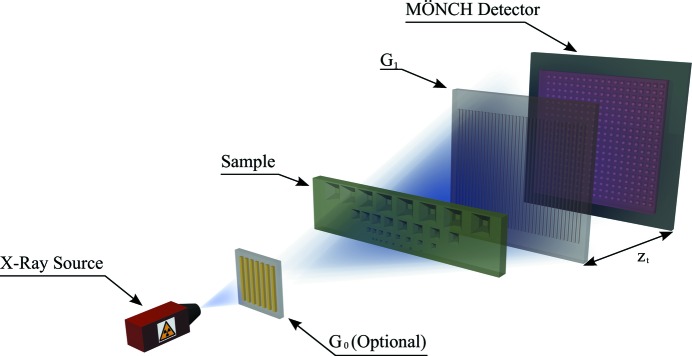
Sketch of the G_2_-less grating interferometer with the MÖNCH hybrid detector. The setup includes an X-ray source (synchrotron or X-ray tube), an optional source grating G_0_ to increase the coherence, the sample, the phase grating G_1_ and the MÖNCH detector which is placed at a Talbot distance 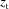 from G_1_.

A sketch of a G_2_-less grating interferometer is illustrated in Fig. 8 ▸. It consists of an X-ray source, either an X-ray tube or a synchrotron beamline, an optional source grating G_0_, a phase grating G_1_ and a MÖNCH hybrid pixel detector. The sample can be placed either between the source and G_1_ or between G_1_ and the detector.

For benchmarking reasons and simplified post-processing due to monochromatic light, the first experiments were performed at the TOMCAT beamline of the Swiss Light Source. However, the method is developed and intended for an X-ray tube-based setup, where the lower flux better matches the requirements for using MÖNCH in the single-photon regime. Additionally, the energy-resolving power of the detector can be exploited for color imaging. The images are acquired at 16.7 keV, using the Si(111) monochromator and additional filters to operate MÖNCH in the single-photon regime. A 4.7 µm-pitch G_1_ silicon phase grating with a duty cycle of 50% and a depth of 33 µm, introducing a phase shift of 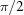, was used for the experiment. The grating was produced at the Laboratory of Micro and Nanotechnology of the PSI.

Compared with the standard Talbot or Talbot–Lau grating interferometer, the analyzer grating G_2_ is not used. The X-ray source illuminates the phase grating G_1_ producing interference fringes at defined distances 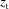 (Talbot distances), where they are detected by MÖNCH.

The experiment consists of three successive measurements with the same exposure time:

(i) *Blank*, using an empty silicon wafer (without grating structures) placed in the beam. It is used to calibrate the interpolation algorithm by calculating the correction map as explained in §4. The empty silicon wafer is used to compensate the photon statistics of the following measurements, preventing extra double counting due to higher flux if no silicon wafer is present.

(ii) *Grating*, taken with only the phase grating G_1_ in the beam and used as a reference for the intensity modulation fringes.

(iii) *Sample*, acquired with both the sample and the grating G_1_ in the beam.

The blank and grating images are required as preparation. They do not contribute to the deposited dose on the sample and they do not need to be repeated for new samples in cases where the setup is unchanged.

### 5.2. Phase retrieval   

Since the G_1_ grating introduces a one-dimension horizontal modulation, after interpolation the intensity is integrated in the vertical direction, parallel to the grating lines within the 25 µm-pitch clusters 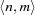 in order to increase the statistics and the visibility of the fringes. Also the differential phase and the absorption signals are retrieved for each pixel cluster 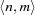. The interpolation technique is used to make the fringes visible, but the granularity of the final image reflects the 25 µm segmentation of the detector.

**Figure 9:**
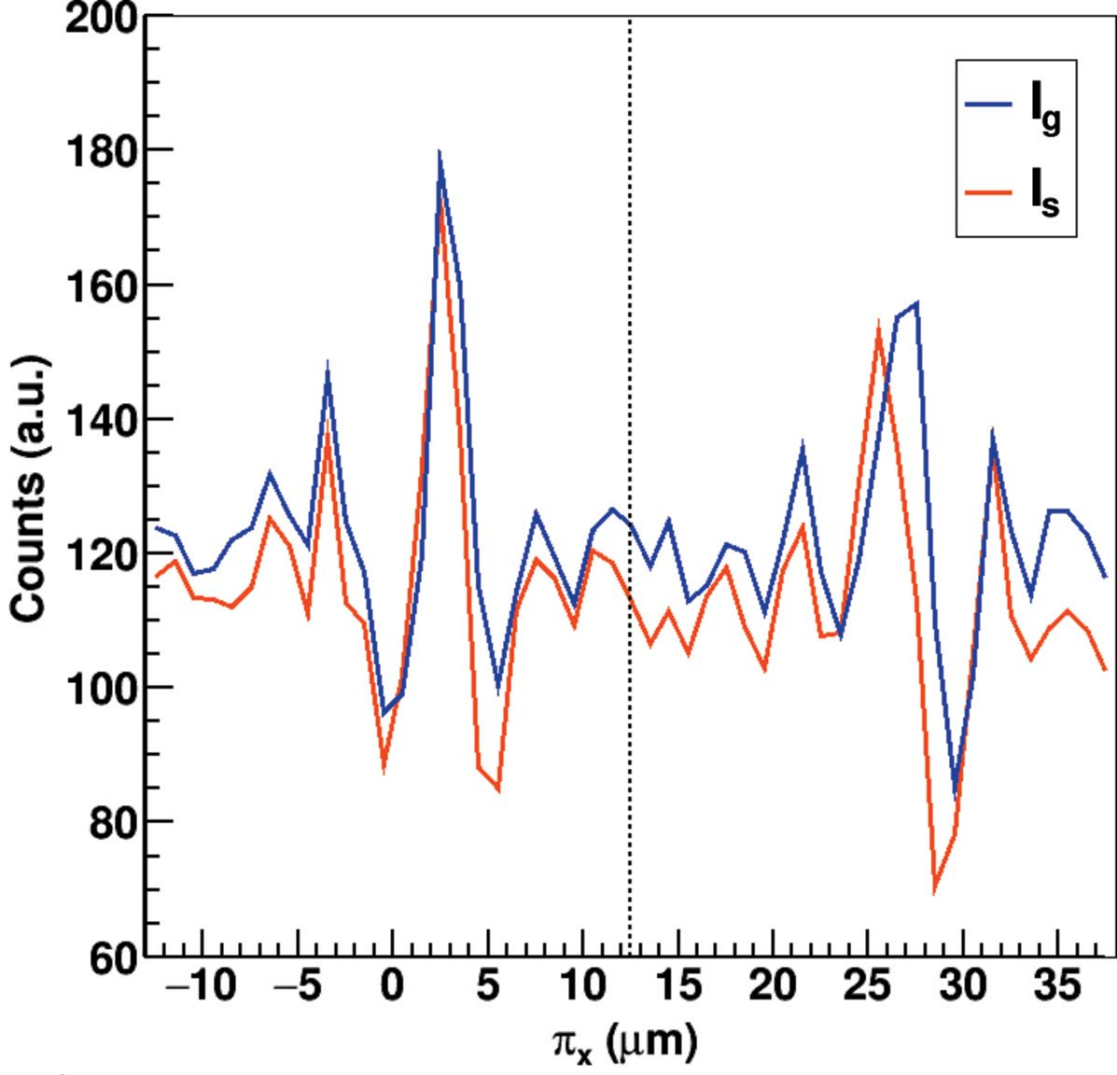
Profile of the grating and sample images of Fig. 11 ▸ for pixel clusters (17127) and (17128) after flat-field normalization. The intensity is integrated in the direction parallel to the gratings. The fringes are visible only in the center of the pixel clusters, *i.e.* at the boundary between two physical pixels, where the spatial resolution is higher. The left-hand pixel is located in a flat region of the sample and therefore shows no phase shift between grating and sample profiles, while the right-hand pixel is located at the pyramid slope and shows a phase shift of about one sub-pixel (1 µm).

In general, the recorded interference fringe can be approximated by a sinusoidal signal, 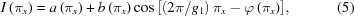
where 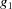 is the period of the recorded interference pattern, 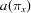 contains the absorption information, 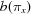 the scattering or visibility reduction but also the response of the pixel cluster due to non-uniform resolution and 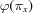 the phase information.


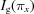
 and 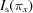 represent the intensity modulation of the grating and of the sample image, respectively, for one of the clusters, where 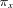 is the horizontal coordinate within the pixel (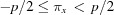). The goal is to retrieve the differential phase contrast (DPC) for each pixel cluster 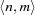, *i.e.* the phase difference between the sample 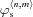 and the grating 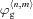 measurements 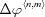 = 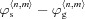. Fig. 9 ▸ shows the profile of the grating and sample images of Fig. 11 for two of the pixel clusters after flat-field normalization. Although the intensity is integrated in the direction parallel to the gratings, the signal remains very noisy due to the low statistics. The fringes are visible only at the center of the pixel clusters, *i.e.* at the boundary between two physical pixels, while their amplitude is dumped close to the center of the physical pixels due to the lower position resolution.

The phase of the sample and grating fringes match for the left pixel cluster, which corresponds to a flat area of the sample, while a shift of approximately one sub-pixel (1 µm) is visible for the right-hand pixel cluster which is located at one of the slopes of the etched pyramid. However, a dedicated technique is necessary in order to correctly retrieve the DPC accounting for the non-uniform resolution of the interpolated signal.

A method based on the Hilbert transform was developed in order to correctly retrieve the DPC, as explained in detail by Kagias *et al.* (2016*a* ▸). The average absorption *A* and differential phase *P* for each pixel cluster can be extracted from the following equations, 
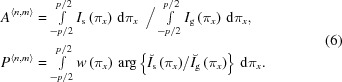

*I*˘_s_(*x*) and *I*˘_g_(*x*) are the analytical signals of the recorded fringes, 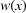 is an appropriate normalized weighting function that accounts for the non-uniform resolution of the interpolation method, and *p* is the pixel pitch. The weighting function is required to enhance the detection of the spatial frequency of the fringes close to the pixel boundaries, while discarding the background given by the photons absorbed in regions where the fringes are not visible, but reduces the dose efficiency of the technique.

In order for the above equations to be applicable, the absorption of the sample should vary slowly within one physical pixel compared with the period of the interference fringe. This is a general requirement for interferometric imaging methods that are based on the direct recording of the interference fringe (Wen *et al.*, 2010 ▸; Bennett *et al.*, 2010 ▸). However, with our method we are able to record fringes with a few micrometers pitch which means that the maximum spatial variations of the sample can be much higher compared with methods utilizing large-pitch gratings.

### 5.3. Imaging results and quantitative validation   

Various samples have been used to demonstrate the validity of the method and also to examine the performance of the imaging technique.

Due to the limited frame rate of the current detector prototype, each of the three images required for the experiment took about 3 h, using a sub-frame exposure time of 12 µs and frame rate of 1 kHz, *i.e.* the sensor is insensitive 99% of the time due to the speed of the current readout electronics. The photon count per physical pixel in the final image was of the order of 25000.

**Figure 10:**
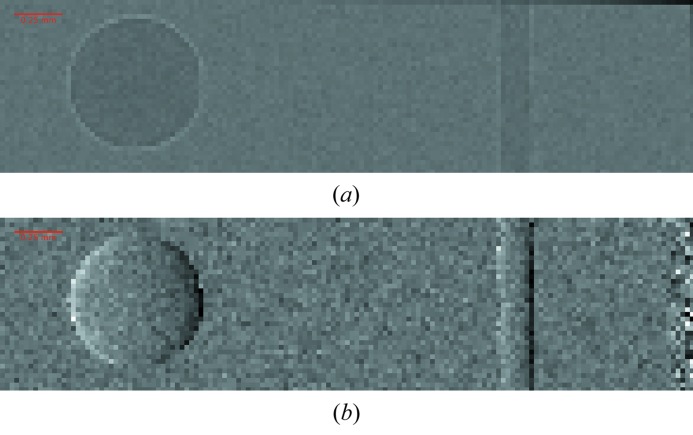
Retrieved (*a*) absorption and (*b*) differential phase contrast images with a pixel size of 25 µm for a polyethylene sphere with 700 µm diameter (left) and a nylon rod with 150 µm diameter (right).

Fig. 10 ▸ shows the absorption and DPC images of a polyethylene sphere with a diameter of 700 µm and a nylon fiber taken from a toothbrush with 150 µm diameter. These samples produce straightforward DPC signals and are therefore easy to interpret.

**Figure 11:**
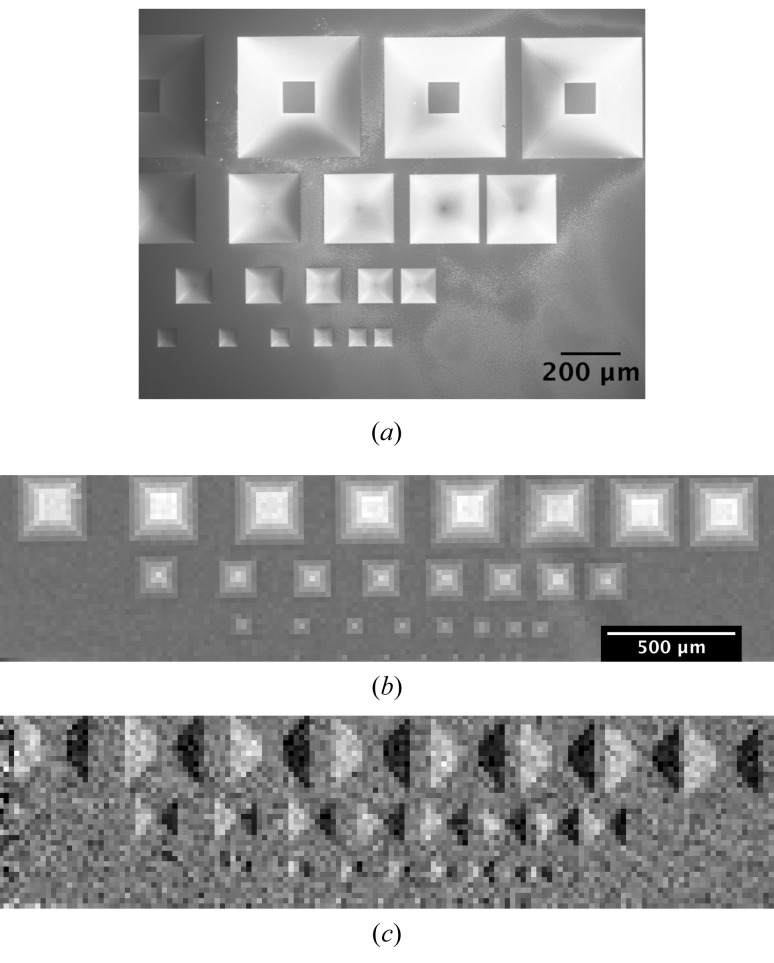
(*a*) SEM image of the pyramid sample. Retrieved (*b*) absorption and (*c*) differential phase contrast images with a pixel size of 25 µm for the pyramids etched in Si.

For a quantitative validation of the technique, pyramids of different sizes etched into a Si substrate were used as a sample. Fig. 11(*a*) ▸ shows a scanning electron microscopy (SEM) image of the sample. The size of the etched pyramids ranges from 50 to 350 µm and the spacing from 200 to 25 µm. It can be observed that the largest pyramids were not etched completely, due to porosity developing in the SiO_2_ mask during the etching which limited the maximum possible etching time. The retrieved absorption and differential phase images are presented in Figs. 11(*b*) and 11(*c*) ▸, respectively. All pyramids are clearly visible in both images, even the smallest one with a size of 50 µm.

The measured differential phase values were used in order to calculate the refraction angles of the detected photons and compared with the theoretical values. The etched pyramids in Si[100] have a slope of ±54.73° which means that 16.7 keV photons impinging at the edges of the pyramids will be refracted by an angle α = 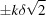 = ±4.9210 µrad, where *k* is the wavenumber and δ the refractive index decrement for Si which at 16.7 keV is 1.7639 × 10^−6^. The measured differential phase value of the slopes is calculated to be *P* = ±(0.4938 ± 0.0869) rad. The refractive angles can be retrieved from the phase difference by α = *P* *g*_1_/2π*z*_t_, where *z*_t_ = 15 cm; from the experimental data this results in α_exp_ = ±(4.9249 ± 0.8664) µrad demonstrating that the quantitative differential phase information is well retrieved.

## 6. Conclusions   

With a pixel size of 25 µm and an extremely low noise of 111 eV r.m.s., the MÖNCH hybrid pixel detector targets low-energy applications where so far only CCDs and CMOS imagers could be used. Moreover, the large amount of charge sharing observable at this small pitch can be exploited to interpolate the hit position of isolated photons, achieving a spatial resolution of the order of 1 µm, as explained in detail in §4. In §5 the high spatial resolution obtained by interpolation is exploited for the direct measurement of the DPC in grating-based phase contrast imaging without the use of the analyzer grating G_2_. This overcomes some of the challenges of grating interferometry which prevent it from moving into clinical practice: low dose efficiency due to the absorption of G_2_, manufacturing limitation for G_2_ in terms of area and aspect ratio (*i.e.* absorption for hard X-rays), long acquisition times due to multiple exposures and complex high-resolution mechanics to perform phase stepping.

However, MÖNCH targets application in many other X-ray experiments both at synchrotrons and using X-ray tubes. The use of dynamic gain switching (Henrich *et al.*, 2011 ▸), which is already implemented in some of the sub-blocks of MÖNCH0.2, will also allow applications at XFELs for soft X-ray beamlines.

In particular, we foresee promising perspectives in soft X-ray applications, for inelastic X-ray scattering or Laue diffraction, where the detector is often the limiting element of the experiment. Compared with the JUNGFRAU 0.4 75 µm-pitch hybrid pixel detector, which already demonstrated a similar noise performance (Jungmann-Smith *et al.*, 2016 ▸), the small pixel pitch of MÖNCH makes it ideal for imaging applications. Despite the larger amount of charge sharing, the interpolation performance decreases at lower energies due to lower SNR. However, we expect to be able to interpolate down to approximately 2 keV with micrometer resolution, which makes it interesting for hard X-ray inelastic X-ray scattering.

For Laue diffraction we expect to be able to determine the energy of single photons with a resolution better than 220 eV r.m.s., improved down to 220 eV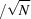 r.m.s. by averaging over the *N* counts in the peak.

Concerning X-ray emission spectroscopy, larger pixels like for JUNGFRAU 0.4 are probably more promising because of the reduced charge sharing. However, the small pixels provided by MÖNCH can increase the maximum detectable flux. Charge sharing can also be suppressed by means of a collimation mask or by excluding the events where charge sharing is observed (software collimation).

### 6.1. Discussion and perspectives   

Despite the promising results, some flaws of the current MÖNCH prototype need to be fixed for improved usage in scientific experiments, in particular for imaging:

(i) *Spatial resolution.* The spatial resolution depends on the photon absorption position within the pixel (higher close to the pixel borders where charge sharing is prominent, lower in the pixel center). An improvement in the resolution could be achieved by utilizing smaller pixels (*e.g.* 20 µm) or by enhancing the charge sharing by increasing the charge collection time. This is obtained by increasing the sensor thickness, reducing the bias voltage or using lower X-ray energies which are absorbed closer to the back-plane of the sensor. The spatial resolution is also affected by the alignment of the sensor relative to the X-ray beam due to the parallax given by the different depth of absorption of the hard X-rays photons through the 320 µm silicon sensor (∼320 nm per 1 mdeg misalignment).

(ii) *Field of view.* The field of view of the the MÖNCH0.2 prototype is limited to 1 mm × 4 mm. Challenges in increasing the detector area include the bump-bonding yield, the maximum frame rate and the data throughput. The larger 1 cm × 1 cm MÖNCH0.3 prototype (160 kpixel) is currently undergoing characterization. The data transfer interface has been upgraded from 1 Gb s^−1^ to 10 Gb s^−1^. It is read out in parallel over 32 analog outputs and can achieve a maximum frame rate of 6 kHz. The huge data throughput of almost 2 GB s^−1^ requires the development of on-the-fly CFA and simultaneous position interpolation. In the future, we plan to design a 3 cm × 2 cm readout ASIC which fully exploits the recticle size offered by the manufacturing process. In the next few years, we plan to build a MÖNCH detector system of 3 cm × 4 cm by tiling together two ASICs sharing the same sensor. The development of the proposed 3 cm × 4 cm detector will deliver a system competitive with CCDs and CMOS imagers for soft X-ray imaging.

(iii) *Measurement time.* With the current frame rate of 1 kHz, single-photon discrimination is possible up to a photon flux of ∼10^5^ photons mm^−1^ s^−1^. At this count rate the acquisition of a high-resolution image with 1 µm^2^ virtual pixel size and a dynamic range of 8-bits (256 counts subpixel^−1^) takes ∼1440 s. However, higher-frequency readout, parallelization and faster data transfer could increase the flux by one to two orders of magnitude and reduce the measurement time to less than 1 min. It has to be noticed that the proposed increase of the detector size will require more sophisticated data acquisition and back-end systems to transfer the data and efficiently store them. Without interpolation, MÖNCH can also operate at higher fluxes, with a frame rate which overtakes the frame rate offered by CCDs and even CMOS imagers by orders of magnitude.

(iv) *Quantum efficiency.* The silicon sensors should be optimized for the detection of soft X-rays below 3 keV which are absorbed in the detector back-plane in the absence of an electric field and therefore cannot be detected. Normally, the aluminium layer can be removed and the thickness of the n^+^-doping modulation can be reduced from micrometers to tens of nanometers by specific doping techniques. The resulting quantum efficiency can improve by almost a factor of two at the silicon *K*-edge (85% absorption efficency for 200 nm-thick back-plane compared with 45% for 1 µm).

Moreover, the 320 µm silicon sensor is relatively transparent for hard X-rays (59% quantum efficiency at 16.7 keV). High-*Z* materials like CdTe or GaAs provide a higher quantum efficiency (both ∼100% quantum efficiency at 16.7 keV and sensor thickness of 500 µm) and can be used for applications up to 100 keV (Steadman *et al.*, 2011 ▸; Hamann *et al.*, 2013 ▸). However, the quality of the high-*Z* sensor materials still needs to be demonstrated and the charge collection performance needs to be characterized in order to properly perform interpolation.

With all the proposed improvements, MÖNCH promises to equal the performance of monolithic detectors or indirect detection systems (scintillators coupled to photodetectors) in many X-ray applications by combining the advantages given by direct conversion, low noise and high resolution with the flexibility given by the well consolidated hybrid technology. Moreover, it will outdo their performance in terms of frame rate.

### 6.2. Single-shot grating interferometry   

The previous discussion concerning the flaws and the possible improvements applies also to the proposed G_2_-less grating interferometry application.

Since the finest fringe period and minimal resolvable phase shift are limited by the ultimate spatial resolution of the detector, currently the fringes are visible only close to the pixel borders and therefore only a fraction of the absorbed photons can be used to retrieve the differential phase information. This is particularly restrictive in medical imaging applications, where the dose delivered to the patient should be limited. Additionally, to improve the operating parameters of the detector by enhancing the charge sharing, the use of a phase grating with a pitch larger than the 4.7 µm currently used would also allow a better exploitation of the position information carried by photons absorbed in the pixel center.

A field of view of a few square centimeters is still insufficient for clinical applications. However, many imaging techniques could already benefit from such a system. In the long term, the use of through silicon vias (TSVs) could allow tiling of ASICs on four sides, aiming at full six-inch wafer sensor assembly with reduced gaps between the ASICs. Similar improvements should follow also concerning the maximum grating area, which is currently limited to four-inch wafers.

Although avoiding phase stepping, the measurement time required in grating interferometry to operate MÖNCH in single-photon regime is still very long. However, the limitation on the maximum detectable flux is striking for direct imaging synchrotron applications but matches well the fluxes of microfocus X-ray tubes. Additionally, the low noise of 31 e^−^ = 110 eV r.m.s. per pixel allows micrometer-resolution color imaging to be performed with an energy resolution of 220 eV r.m.s. on the 2 × 2 pixel cluster.

An improvement of the quantum efficiency for hard X-rays is extremely important for grating interferometry and medical examinations in general, since it brings a proportional reduction of the dose.

Future work will also involve the replacement of the one-dimensional phase grating with a two-dimensional periodic structure. This can allow the retrieval of quantitative differential phase in two directions and can be used to obtain quantitative phase values without integration artifacts (Kottler *et al.*, 2007 ▸; Kagias *et al.*, 2016*b* ▸).

All these improvements will result in significant steps towards a broad implementation of phase contrast imaging in the medical field and beyond.
